# Development of a brief screening measure of unmet supportive care needs (SCNS-P&C-6) in caregivers of people with high-grade glioma

**DOI:** 10.1186/s41687-024-00835-4

**Published:** 2025-01-10

**Authors:** Jill Chen, Joanne M. Shaw, Haryana M. Dhillon, Georgia K. B. Halkett, Emma McDougall, Anna K. Nowak, Rachel Campbell, for the BRAINs Program Investigators

**Affiliations:** 1 Psycho-Oncology Cooperative Research Group, School of Psychology, Faculty of Science, The University of Sydney, Camperdown, NSW 2006 Australia; 2 Curtin School of Nursing/Curtin Health Innovation Research Institute, Faculty of Health Sciences, Curtin University, Bentley, WA 6102 Australia; 3 Medical School, The University of Western Australia, Crawley, WA 6009 Australia; 4 Department of Medical Oncology, Sir Charles Gairdner Hospital, Nedlands, WA 6010 Australia

**Keywords:** Brain cancer, Caregiver, Screening, Unmet needs, Supportive care

## Abstract

**Purpose:**

Informal caregivers of people with high grade glioma (HGG) often have high levels of unmet support needs. Routine screening for unmet needs can facilitate appropriate and timely access to supportive care. We aimed to develop a brief screening tool for HGG caregiver unmet needs, based on the Supportive Care Needs Survey—Partners & Caregivers (SCNS-P&C).

**Methods:**

Secondary analysis was performed on responses to the SCNS-P&C from 188 HGG caregivers, who participated in the Care-IS trial. SCNS-P&C items were assessed against four criteria: factor loadings; prevalence; variation in domain score; diagnostic accuracy. Supplementary analysis was conducted at two timepoints (T1 & T2) on the final selected items to identify caregivers indicating no needs on the screening items but reported a need on the original SCNS-P&C, suggesting they would be “missed” by the screening items.

**Results:**

Six items performed best against psychometric criteria, capturing two domains: *Cancer impact needs* and *Information and communication needs*. Supplementary analysis showed screening items failed to identify only 7.4% (14/188) of caregivers with other unmet needs at T1 and 11.4% (18/158) at T2. Of those missed at T1, only four were missed again at T2.

**Conclusions:**

We identified six-items for inclusion in a brief screening tool, the SCNS-P&C-6, demonstrating good sensitivity in detecting unmet needs of caregivers of people with HGG. Use of this tool in clinical practice has the potential to improve access to care and the cancer experience for both the caregiver and person with brain tumor.

**Supplementary Information:**

The online version contains supplementary material available at 10.1186/s41687-024-00835-4.

## Abstract (plain language summary)

Family members or friends who look after people with brain tumors often have supportive care needs that are inconsistently identified and not well addressed by health systems. This study sought to create a short screening measure that can be used in clinical practice settings to identify people in these caregiving roles who need additional support. We identified six questions that can be administered as a short screening questionnaire that are likely to identify most people caring for people with brain tumor with unmet supportive care needs. This screening tool has potential to be used in clinical practice to identify caregivers requiring additional support, triggering provision of targeted resources and support services.

**Supplementary Information:**

The online version contains supplementary material available at 10.1186/s41687-024-00835-4.

## Introduction

“Brain and CNS cancer” refers to different malignant tumors occurring in the brain and associated tissue, characterized by brain site and histology [1]. High grade glioma (HGG) is the most common presentation of brain tumor and people living with HGG (PwHGG) experience a range of symptoms and changes due to the disease and its treatment. These can include fatigue, disturbed sleep, pain, nausea, personality changes, and cognitive impairment [2–4]. Due to these complex symptoms and their functional impacts, PwHGG often rely on informal caregivers (i.e., family or friends) for support. In turn, caregivers commonly find supporting people with HGG challenging, particularly due to the poor prognosis accompanied by their rapid deterioration in health [3].

The complexity of patient care and ongoing high level of impairment means caregivers of PwHGG have high levels of supportive care needs [5, 6]. Halkett et al. found up to 80% of family caregivers of PwHGG had at least one moderate or high unmet need and 59% five unmet needs [5]. Higher unmet needs are associated with increased distress, feelings of anxiety and depression, lower confidence in care, and lower preparedness to care, particularly during treatment [5–8]. Frequently reported needs unique to the brain tumor experience include needing information on adjusting to cognitive changes, managing difficult aspects of the patient’s behavior, and adjusting to changes in the patient’s personality [5]. More generally, caregivers of PwHGG consistently report needs relating to communication with health professionals, information, service provision, and psychological and social support [9]. Caregiver needs change over time, while psychological distress and morbidity remain consistently high throughout the disease trajectory [5, 10].

Caregivers typically face a lack of support within healthcare systems, with only generic non-brain cancer specific psychological support available via the community [11, 12]. Globally, caregivers of people with brain tumor report similar experiences, challenges, and unmet needs [13]. Routine screening for unmet need among caregivers should facilitate appropriate and timely access to supportive care [10, 14, 15]. The **B**rain cancer **R**ehabilitation, **A**ssessment, and **I**nterventions for Survivorship **N**eed**S** (BRAINS) program has expanded an existing online screening and referral portal [16] to incorporate a clinical pathway for identification and management of unmet needs among people with brain tumor (PwBT), the ADAPT BRAINS portal. ADAPT BRAINS incorporates screening for unmet needs, anxiety, and depression, recommending appropriate management strategies based on a stepped-care model [17]. This portal and clinical pathway is being further adapted for caregivers of PwBT.

There is currently no fit-for-purpose caregiver unmet needs measure that could be implemented as a brief screening tool in the portal or clinical practice settings more broadly. Existing measures are lengthy (> 20 items) [18, 19], with implications for caregiver time and respondent burden, and are unlikely to be feasible for use in routine care [20, 21]. To our knowledge, only two unmet needs measures targeting brain cancer caregivers exists, the Caregiver Needs Screen [22] and a measure developed by Parvataneni et al. [23], both of which have limitations. Specifically, more than half of the items in the Caregiver Needs Screen assess concerns related to the patient’s health rather than caregivers’ own wellbeing and needs, possibly perpetuating caregiver’s tendencies to neglect their own needs. Moreover, the Parvataneni et al. measure is not psychometrically validated and both measures are lengthy (> 20 items), limiting their feasibility for routine use in clinical practice. One widely used, validated measure is the 44-item Supportive Care Needs Survey—Partners & Caregivers (SCNS-P&C) [24]. The SCNS-P&C was developed through adaption of the original patient version of the SCNS, following review of the literature and existing unmet needs measures, stakeholder feedback, and psychometric validation. The SCNS-P&C is intended to be a comprehensive, multi-dimensional assessment of caregiver unmet needs across the cancer illness trajectory. We aimed to develop a brief screening measure based on the SCNS-P&C to identify caregivers with unmet needs relating to caring for someone with a brain tumor and facilitate appropriate triaging for support.

## Methods

### Sample

Secondary analysis was performed on data collected from 188 caregivers of patients with Grade III or IV HGG who participated in the Care-IS randomized controlled trial [25, 26]. Care-IS, a nurse-led supportive educational intervention for caregivers of PwHGG, aimed to improve preparedness to care and reduce caregiver distress. Eligibility criteria included: being a caregiver of a PwHGG undergoing active treatment, within two months of initial diagnosis, aged 18 or above, with functional English language skills. Participants were recruited from eight Australian sites: three in Perth, Western Australia; and five in Sydney, New South Wales. All participants provided informed consent. Recruitment was conducted between February 2014 and June 2019. The trial protocol detailing the methods and the main study results are reported elsewhere [25, 26]. Ethical approval for the Care-IS trial was obtained from all sites (NSW: HREC 16/105; SJOG: 671; SCGH: 2013 − 172; Curtin University: HR 17/2013). Trial registration number: Australian and New Zealand Clinical Trials Registration (ACTRN) 12612001147875.

### Measures

Care-IS trial participants completed measures at baseline and follow-up time points [25, 26]. Paper-based surveys were completed at home and returned via mail. These surveys included demographic questions and the SCNS-P&C.

Demographic information collected included participants’ age, gender, length of caregiving, relationship to the PwHGG, marital status, place of birth, language spoken at home, number of children, and education level.

The SCNS-P&C [24] is a 44-item measure assessing cancer caregivers’ unmet needs. Factor analysis identified four domains: *Health Care Service Needs*,* Psychological and Emotional Needs*,* Work and Social Needs*, and *Information Needs*, with reliability for each domain ranging from good to excellent (*α* = 0.88-0.94). Each item is preceded with the stem question, ‘In the last month, what was your level of need for help with…’. Items are rated on a five point scale, ranging 1–5. Response options are: (1) ‘No need—Not applicable,’ (2) ‘No need, satisfied,’ (3) ‘Some need—Low need,’ (4) ‘Some need—Moderate Need,’ (5) ‘Some need—High need.’ Scoring involves summing item scores within each domain and transforming summed scores into a 0-100 standardised score [27].

### Statistical analysis plan

The analyses conducted were adapted from an approach published by Girgis et al. to develop a brief screening measure of patients’ unmet needs [28]. Our main psychometric analyses focused on caregiver baseline data collected prior to randomization to the Care-IS intervention arm or usual care. Supplementary analysis was conducted using baseline (T1) and week 8 (T2) responses to the SCNS-P&C.

We first conducted item level analysis of the SCNS-P&C, beginning with exploratory factor analysis (EFA) using principal components extraction and an orthogonal (varimax) rotation. Following the EFA, frequency analysis and simple linear regressions were used to determine item prevalence and variation in the domain score explained by single items, respectively. Two- and three-item combinations were generated after analysis of single items. Simple linear regressions and receiver operating curve (ROC) analysis (used to determine diagnostic accuracy) were conducted using these item combinations. Results of these analyses were assessed against key psychometric criteria to determine the final items to include in the brief screening measure. Below, our data analysis strategy and criteria are outlined in detail.

### Criteria for item selection

#### Step 1: single item analyses

**Criterion 1: Factor loadings**. Items with larger factor loadings on their respective domains were considered superior in predicting that unmet need domain score [28]. The number of factors extracted was based on evaluation of Kaiser’s criterion of eigenvalues ≥ 1 [29], examination of the scree plot, and parallel analysis [30]. Items were retained if they exhibited a factor loading ≥ 0.30 and did not cross-load onto other factors (a cross-loading difference of 0.05 was used as a cut-off value) [31]. Only items retained in the EFA were further assessed as potential screening items in subsequent analyses.

**Criterion 2: item prevalence**. Item responses were dichotomized into ‘no need’ (no need—not applicable, need satisfied; scores 1–2) or ‘some need’ (low, medium, or high need; scores 3–5). The proportion (%) of caregivers reporting any level of need (i.e., low, moderate, or high) was calculated for each item retained by the EFA. Priority was given to items that showed a higher proportion of some level of need.

**Criterion 3: variation in the unmet need domain score**. Each item’s raw score was regressed against their corresponding standardized domain score (0-100), excluding that item. The magnitude of the coefficient of determination (*R*^*2*^) was reported and compared between potential screening items. Items explaining greater variation in the domains extracted by EFA were considered superior in predicting the domain score [28].

#### Step 2: analyses examining two- and three-item combinations

For each unmet need domain (as determined by EFA), the 10 items performing best across criteria 1–3 were selected and combined to form all possible two- and three-item combinations. The variation in each domain score explained by the two- and three-item combinations (i.e., Criterion 3) was assessed for each possible combination. In these analyses, each two- and three-item combination was regressed against their respective domain scores, excluding the regressed items from that domain score (i.e., two or three potential screening items). Items deemed too similar in content were not included in the same combinations to avoid redundant items in the final screening measure. For example, items 1 (*accessing information relevant to your needs as a carer/partner*) and 3 (*accessing information about support services for carers/partners of people with cancer*) are similar in content as they both capture information needs specific to supporting the caregiver, and so were not included in a combination together.

**Criterion 4: diagnostic accuracy**. For each unmet need domain, the 10 best performing two- and three-item combinations were selected and assessed for their diagnostic accuracy. Specifically, ROC analysis was performed to determine the accuracy of each item combination in discriminating between caregivers who had *any* unmet need versus those with *no* unmet need in each domain. The dependent variable in these analyses was a binary outcome (excluding items of interest), where 0 indicated no need in the given domain and 1 indicated any need was present. The independent variable (test variable) in each analysis was all possible two- and three-item combinations. Each item combination was summed to generate a standardized continuous score ranging between 0 and 100. An area under the curve (AUC) ≥ 0.80 indicates excellent ability in discriminating between caregivers with and without unmet needs [32]. Item combinations with higher AUC values were deemed better candidates for inclusion in the final screening measure.

ROC analysis also provides sensitivity and 1-specificity results. Sensitivity refers to the proportion of individuals *correctly* identified as having a need. 1-specificity refers to the proportion of people *incorrectly* identified as having a need. As SCNS-P&C is not a clinical measure and does not have a clinical cut-off value, a non-clinical cut-off was identified for sensitivity and 1-specificity to estimate the extent to which each domain can correctly identify those with any unmet need balanced against extent of incorrect identification.

The final screening items were selected based on item combinations that performed best against Criteria 3 & 4, for each domain. Individual item results were also considered (i.e., factor score and item prevalence; Criterion 1 & 2) as well as the content captured by each item to aid decision making. Item content that targeted needs focused on the caregiver, rather than the caregiver meeting patient needs, was preferred as the intention was to create a screening tool indicative of caregiver-centered support needs. Authors JC, RC, JS, and HD were involved in the decision-making process.

### Supplementary analysis: proportion (%) of individuals missed by final items selected for inclusion in the brief screening measure

Supplementary analysis was conducted at two time points using the final items selected for inclusion in the brief screening measure to determine what proportion of caregivers with other unmet needs were not detected by the selected screening items. Frequency analysis, conducted on T1 and T2 responses to the 44-item SCNS-P&C, determined the proportion (%) of individuals with other unmet needs of any level (low, moderate, and high) who were not identified as having a need by the final items selected for inclusion, and were therefore “missed” by the brief screening measure [33].

## Results

Demographics of the 188 caregivers are presented in Table 1. Participants’ mean age was 57 years (SD = 11.6, range = 23–85). Most (74.5%) caregivers were female and 87.8% were partners of patients. Mean length of caregiving was 3 months (SD = 6.8, range = 0.50–84).

**Table 1:** Characteristics of caregivers (total *N* = 188)

Caregiver characteristics	*N* (%)
Gender	
Male	48 (25.5)
Female	140 (74.5)
Length of caregiving (*N =* 174*)*	
< 1 month	3 (1.7)
1–3 months	142 (81.6)
3–6 months	21 (12.1)
6–12 months	5 (2.9)
> 12 months	3 (1.7)
Relationship to patient	
Spouse/Partner	165 (87.8)
Other (e.g. parent, child)	23 (12.2)
Marital status	
Married/partner	173 (92)
Widowed	1 (0.5)
Divorced/separated	3 (1.6)
Never married	11 (5.9)
Place birth (*N* = 187)	
Australia	113 (60.4)
New Zealand	9 (4.8)
Fiji	1 (0.5)
Europe	44 (23.5)
Asia	10 (5.3)
Africa	6 (3.2)
North America	4 (2.1)
South America	1 (0.5)
English spoken at home (*N* = 186)	
Yes	172 (92.5)
No	14 (7.5)
Number of children (*N =* 186)	
0	25 (13.4)
1–2	101 (54.3)
3–4	51 (27.4)
> 4	9 (4.8)
Number of children at home (*N* = 167)	
0	104 (62.3)
1–2	49 (29.3)
3–4	14 (8.4)
Education (*N* = 186)	
High school or below	50 (26.9)
Postsecondary education	136 (73.1)
Current employment status (*N =* 186)	
Full-time employed	61 (32.8)
Part-time employed	23 (12.4)
Unemployed	12 (6.5)
Retired	53 (28.5)
Other^a^	37 (19.9)
Financial effect of diagnosis (*N* = 186)	
Had no effect on my financial situation	46 (24.7)
Had a slight effect on me financially	78 (41.9)
Had a significant effect on me financially	56 (30.1)
Other^b^	6 (3.2)

^a^‘Other’ allowed for an open-ended response. Responses included being on leave, self-employed, on disability pension, casual, carer, homemaker, and student

^b^‘Other’ allowed for an open-ended response. Responses detailed not being financially affected at the time but anticipating future financial effects

### Single item analyses

**Criterion 1: factor loadings**. EFA identified two factors with eignvalues > 1, accounting for 47% of the variance (Kaiser-Meyer-Olkin test = 0.90; Bartlett’s test *p* = < 0.001). Parrallel analysis also indicated a 2-factor solution. The two identified factors or “unmet need domains” captured were: *Cancer impact needs* and *Information and communication needs*. Twenty-two items loaded onto *Cancer impact needs* and 15 items loaded onto *Information and communication needs*. Factor loadings ranged from 0.41 to 0.85 (see Table 2 for final factor loadings). In total, 37 items were retained by EFA and assessed in subsequent analyses; seven items were excluded due to factor loadings < 0.30 or cross-loadings of > 0.05 (see Supplementary File 1 for full EFA results).

**Criterion 2: item prevalence**. Prevalence of caregivers reporting an item as an unmet need ranged from 16 to 62% (see Table 2 for prevalence of all items). The four most common needs were items 42 (*making decisions about your life in the context of uncertainty*), 22 (*the impact that caring for the person with cancer has had on your working life*,* or usual activities*), 39 (*working through your feelings about death and dying*) and 2 (*accessing information about the person with cancer’s prognosis*,* or likely outcome*). The three least common needs were items 43 (*exploring your spiritual beliefs*), 27 (*communicating with the family*) and 16 (*obtaining adequate pain control for the person with cancer*).

**Criterion 3: variation in the unmet needs domain score**. The variance explained by each corresponding item for the *Cancer impact needs* domain ranged from 0.12 to 0.56, and for the *Information and communication needs* domain ranged from 0.37 to 0.61. The three items accounting for the most variance in the *Cancer impact needs* domain were items 37 (*getting emotional support for yourself*), 34 (*balancing the needs of the person with cancer and your own needs*), and 42 (*making decisions about your life in the context of uncertainty*). The three items accounting for the most variance in the *Information and communication needs* domain were items 9 (*being involved in the person with cancer’s care*,* together with the medical team*), 10 (*having opportunities to discuss your concerns with the doctors*), and 11 (*feeling confident that all the doctors are talking to each other to coordinate the person with cancer’s care*).

**Table 2:** 37 single items factor loadings, prevalence, and coefficient of determination (*R*^*2*^)

Item No.	Item	Factor loading	Prevalence (%)	*R* ^2^
*Domain 1: Cancer impact*,* understanding*,* and support needs*				
15	Looking after your own health, including eating and sleeping properly	0.65	46.28	0.40
21	Adapting to changes to the person with cancer’s working life, or usual activities	0.64	50.53	0.43
**22**	**The impact that caring for the person with cancer has had on your working life**,** or usual activities**	**0.69**	**60.11**	**0.45**
23	Finding out about financial support and government benefits for you and/or travel insurance for the person with cancer	0.53	58.51	0.35
26	Communicating with the person you are caring for	0.58	22.34	0.37
27	Communicating with the family	0.55	16.49	0.31
28	Getting more support from your family	0.45	19.15	0.15
29	Talking to other people who have cared for someone with cancer	0.44	44.68	0.24
30	Handling the topic of cancer in social situations or at work	0.48	29.26	0.22
32	The impact that cancer has had on your relationship with the person with cancer	0.75	36.70	0.50
**33**	**Understanding the experience of the person with cancer**	**0.67**	**55.32**	**0.44**
34	Balancing the needs of the person with cancer and your own needs	0.76	55.85	0.55
35	Adjusting to changes in the person with cancer’s body	0.54	42.02	0.32
36	Addressing problems with your sex life	0.41	26.06	0.12
**37**	**Getting emotional support for yourself**	**0.75**	**55.32**	**0.56**
38	Getting emotional support for your loved ones	0.65	52.66	0.40
39	Working through your feelings about death and dying	0.64	59.04	0.44
40	Dealing with others not acknowledging the impact on your life of caring for a person with cancer	0.68	46.28	0.40
41	Coping with the person with cancer’s recovery not turning out the way you expected	0.55	44.68	0.31
42	Making decisions about your life in the context of uncertainty	0.70	61.70	0.53
43	Exploring your spiritual beliefs	0.57	15.96	0.29
44	Finding meaning in the person with cancer’s illness.	0.58	35.64	0.28
*Domain 2: Information and communication needs*				
**1**	**Accessing information relevant to your needs as a carer/partner**	**0.59**	**48.40**	**0.37**
2	Accessing information about the person with cancer’s prognosis, or likely outcome	0.55	59.04	0.31
3	Accessing information about support services for carers/partners of people with cancer	0.56	51.60	0.37
**5**	**Accessing information on what the person with cancer’s physical needs are likely to be**	**0.62**	**55.32**	**0.39**
6	Accessing information about the benefits and side-effects of treatments so you can participate in decision making about the person with cancer’s treatment	0.68	52.13	0.47
7	Obtaining the best medical care for the person with cancer	0.77	42.02	0.51
8	Accessing local health care services when needed.	0.69	36.17	0.48
9	Being involved in the person with cancer’s care, together with the medical team	0.85	36.17	0.61
**10**	**Having opportunities to discuss your concerns with the doctors**	**0.80**	**45.74**	**0.59**
11	Feeling confident that all the doctors are talking to each other to coordinate the person with cancer’s care	0.80	41.49	0.54
12	Ensuring there is an ongoing case manager to coordinate services for the person with cancer	0.77	42.02	0.52
13	Making sure complaints regarding the person with cancer’s care are properly addressed	0.74	27.66	0.50
14	Reducing stress in the person with cancer’s life	0.61	56.91	0.43
16	Obtaining adequate pain control for the person with cancer	0.52	18.09	0.23
17	Addressing fears about the person with cancer’s physical or mental deterioration	0.55	55.32	0.35

*Note* Final screening items are bolded

### Analyses examining two- and three-item combinations

Item selection for further analyses was based on assessment of the best 10 performing against *all three criteria* (i.e., criteria 1–3). Within the *Cancer impact needs* domain, items 15, 21, 22, 33, 34, 37, 38 and 42 were selected (see Table 2 for item content). Within the *Information and communication needs* domain, items 5, 6, 7, 10, 12, 14 were selected for further analyses (see Table 2 for item content). Items 1 (*accessing information relevant to your needs as a carer/partner*) and 3 (*accessing information about support services for carers/partners of people with cancer*), which were two of the 10 most prevalent items within the *Information and communication needs* domain (reported by 48% and 52% of caregivers, respectively), were also selected for inclusion in further analyses as they targeted needs focused on the caregiver rather than the patient. We did not conduct analyses on combinations featuring items 1 and 3 together to avoid redundancy in the final measure. We also did not conduct analyses on combinations featuring items 5 (*accessing information on what the person with cancer’s physical needs are likely to be)* and 6 (*accessing information about the benefits and side-effects of treatments so you can participate in decision making about the person with cancer’s treatment)* together, as both captured information needs specific to patient physical wellbeing.

### Cancer impact needs domain

Twenty-eight two-item combinations and 56 three-item combinations were generated from selected items. Overall, *R*^*2*^ for all possible two-item combinations (range = 0.45-0.68) were lower than for all possible three-item combinations (range = 0.56-0.74). Therefore, we selected the 10 three-item combinations with the highest *R*^*2*^ for ROC analysis. See Supplementary File 2 for regression results for all two-item combinations.

Table 3 summarises the *R*^*2*^ and AUCs of the 10 best performing three-item combinations. Items 22 (*The impact that caring for the person with cancer has had on your working life*,* or usual activities)*, 33 (*Understanding the experience of the person with cancer*) and 37 (*Getting emotional support for yourself*) were considered superior to any other item pair, triplet, or single item. Individually, they had high factor loadings (0.69, 0.67, 0.75, respectively), and more than half of participants indicated some level of need on each item (60%, 55%, 55%, respectively). Together, they explained the most variance in the *Cancer Impact* domain (*R*^*2*^ = 0.74), and demonstrated excellent discrimination (AUC = 0.91; Table 3). Of the caregivers who have some level of need on these three items, 86% would likely be correctly identified as having a need (see Supplementary File 4 for sensitivity and 1-specificity results). Thus, these three items were selected to capture *Cancer impact needs* in the final screening measure.

### Information and communication needs domain

Twenty-six two-item combinations and 44 three-item combinations were generated from selected items. For the information needs domain, *R*^*2*^ for all possible two-item combinations (range = 0.47-0.65) were generally lower than for three-item combinations (range = 0.50-0.74). Again, we selected the 10 best performing three-item combinations with the highest *R*^*2*^ for further analyses. See Supplementary File 3 for the regression results for all two-item combinations.

Items 1 (*Accessing information relevant to your needs as a carer/partner*), 5 (*Accessing information on what the person with cancer’s physical needs are likely to be*), and 10 (*Having opportunities to discuss your concerns with the doctors*) were considered superior to other item pairings, triplets, or single items. Individually, they had good to strong factor loadings (0.59, 0.62, 0.80, respectively), and nearly half of all participants indicated some level of need for each item (48%, 55%, 46%). Together, they explained the most variance in the *Information and communication* domain (*R*^*2*^ = 0.74) and demonstrated excellent discrimination (AUC = 0.92; Table 3). Of the caregivers who have some level of need on these three items, 85% would likely be correctly identified as having a need (See Supplementary File 4 for sensitivity and 1-specificity results). Thus, items 1, 5 and 10 were selected to capture information and communication needs in the final screening measure.

**Table 3:** Three-item combinations *R*^*2*^ and AUC

Item 1	Item 2	Item 3	*R* ^2^	AUC
*Domain 1: Cancer impact needs*				
**Item 22 The impact that caring for the person with cancer has had on your working life**,** or usual activities**	**Item 33 Understanding the experience of the person with cancer**	**Item 37 Getting emotional support for yourself**	**0.74**	**0.91**
Item 22 The impact that caring for the person with cancer has had on your working life, or usual activities	Item 33 Understanding the experience of the person with cancer	Item 38 Getting emotional support for your loved ones	0.71	0.88
Item 22 The impact that caring for the person with cancer has had on your working life, or usual activities	Item 38 Getting emotional support for your loved ones	Item 42 Making decisions about your life in the context of uncertainty	0.71	0.94
Item 21 Adapting to changes to the person with cancer’s working life, or usual activities	Item 34 Balancing the needs of the person with cancer and your own needs	Item 42 Making decisions about your life in the context of uncertainty	0.71	0.89
Item 15 Looking after your own health, including eating and sleeping properly	Item 22 The impact that caring for the person with cancer has had on your working life, or usual activities	Item 33 Understanding the experience of the person with cancer	0.70	0.91
Item 15 Looking after your own health, including eating and sleeping properly	Item 34 Balancing the needs of the person with cancer and your own needs	Item 38 Getting emotional support for your loved ones	0.70	0.91
Item 22 The impact that caring for the person with cancer has had on your working life, or usual activities	Item 37 Getting emotional support for yourself	Item 38 Getting emotional support for your loved ones	0.70	0.91
Item 21 Adapting to changes to the person with cancer’s working life, or usual activities	Item 33 Understanding the experience of the person with cancer	Item 37 Getting emotional support for yourself	0.70	0.91
Item 22 The impact that caring for the person with cancer has had on your working life, or usual activities	Item 33 Understanding the experience of the person with cancer	Item 34 Balancing the needs of the person with cancer and your own needs	0.70	0.86
Item 34 Balancing the needs of the person with cancer and your own needs	Item 38 Getting emotional support for your loved ones	Item 42 Making decisions about your life in the context of uncertainty	0.70	0.93
*Domain 2: Information and communication needs*				
**Item 10 Having opportunities to discuss your concerns with the doctors**	**Item 5 Accessing information on what the person with cancer’s physical needs are likely to be**	**Item 1 Accessing information relevant to your needs as a carer/partner**	**0.74**	**0.92**
Item 12 Ensuring there is an ongoing case manager to coordinate services for the person with cancer	Item 6 Accessing information about the benefits and side-effects of treatments so you can participate in decision making about the person with cancer’s treatment	Item 3 Accessing information about support services for carers/partners of people with cancer	0.72	0.88
Item 12 Ensuring there is an ongoing case manager to coordinate services for the person with cancer	Item 6 Accessing information about the benefits and side-effects of treatments so you can participate in decision making about the person with cancer’s treatment	Item 14 Reducing stress in the person with cancer’s life	0.71	0.89
Item 10 Having opportunities to discuss your concerns with the doctors	Item 6 Accessing information about the benefits and side-effects of treatments so you can participate in decision making about the person with cancer’s treatment	Item 3 Accessing information about support services for carers/partners of people with cancer	0.71	0.89
Item 7 Obtaining the best medical care for the person with cancer	Item 5 Accessing information on what the person with cancer’s physical needs are likely to be	Item 1 Accessing information relevant to your needs as a carer/partner	0.70	0.90
Item 12 Ensuring there is an ongoing case manager to coordinate services for the person with cancer	Item 6 Accessing information about the benefits and side-effects of treatments so you can participate in decision making about the person with cancer’s treatment	Item 1 Accessing information relevant to your needs as a carer/partner	0.70	0.88
Item 10 Having opportunities to discuss your concerns with the doctors	Item 7 Obtaining the best medical care for the person with cancer	Item 3 Accessing information about support services for carers/partners of people with cancer	0.69	0.88
Item 12 Ensuring there is an ongoing case manager to coordinate services for the person with cancer	Item 7 Obtaining the best medical care for the person with cancer	Item 3 Accessing information about support services for carers/partners of people with cancer	0.69	0.87
Item 10 Having opportunities to discuss your concerns with the doctors	Item 5 Accessing information on what the person with cancer’s physical needs are likely to be	Item 3 Accessing information about support services for carers/partners of people with cancer	0.69	0.89
Item 12 Ensuring there is an ongoing case manager to coordinate services for the person with cancer	Item 5 Accessing information on what the person with cancer’s physical needs are likely to be	Item 1 Accessing information relevant to your needs as a carer/partner	0.69	0.90

*Note* Final screening items are bolded

### Supplementary analysis: proportion (%) of individuals with unmet needs missed

At T1, 14 of 188 participants (7.4%) with other unmet needs on the full 44-item SCNS-P&C were missed by the six screening items selected for inclusion in the final brief screening version of the measure. Of the 14 missed, three had high needs. These were: *‘Accessing information about the benefits and side-effects of treatments so you can participate in decision making about the person with cancer’s treatment;’ ‘Finding out about financial support and government benefits for you and/or travel insurance for the person with cancer;’* and *‘Potential fertility problems in the person with cancer.’*

At T2, 18 of 158 participants (11.4%) with other unmet needs were missed by the six screening items. A single missed caregiver had a high need: *‘Finding out about financial support and government benefits for you and/or travel insurance for the person with cancer.’*

Of the 14 participants missed at T1, seven were detected as having a need at T2. Of the seven missed again at T2, three had not completed the SCNS-P&C. For more detail on the severity and type of needs reported by individuals missed at both timepoints, see Supplementary File 5.

## Discussion

From the original SCNS-P&C, we identified six items for inclusion in a brief tool designed to screen for unmet needs of caregivers of PwHGG, the SCNS-P&C-6. The items capture two domains related to cancer impacts and information and communication. Supplementary analysis showed 7.4% of the sample were “missed” by the six screening items at T1, and 11.4% at T2. Four of the caregivers missed at T1 were missed again at T2. Our findings support the potential clinical utility of this tool for screening purposes and supplementary analyses underscore the importance of regular re-screening as part of long-term follow up to ensure individuals missed previously are identified subsequently. See the Appendix for the SCNS-P&C-6.

Caregivers of PwHGG have expressed multi-faceted, complex impacts on their own lives, emotional wellbeing, and health on undertaking a caring role [5, 8]. Often caregivers reduce work outside the home to meet the care needs of the PwHGG [13, 34]. Conflicts may arise between care demands and pre-existing roles, such as caring for children or aged parents [35]. Such disruptions to their daily lives can lead to their feeling isolated from social networks, contributing to distress. Caregivers often seek information and support from health professionals to help with their responsibilities, and a lack of such support can heighten feelings of depression and anxiety [9, 13]. These impacts can also be experienced among caregivers of PwBT more generally [2, 13, 36]. Combined with a strong desire to protect and prioritize the needs of the PwHGG, caregivers are unlikely to seek support for their own needs, a problem exacerbated by the lack of screening and formal care pathways in the health system for informal caregivers [3, 13].

To overcome these difficulties, a clinical pathway addressing distress and unmet needs in caregivers of PwBT, running alongside but separate to a patient clinical pathway, is needed. The ADAPT BRAINS Portal seeks to fill this need in Australia. The present analysis supports incorporation of the SCNS-P&C-6 into the ADAPT BRAINS Portal as part of a caregiver screening pathway. To ensure screening questions are suitable for use with caregivers of PwBT in general, we will pilot the tool with these caregivers and explore its feasibility and acceptability. Pilot testing will identify items potentially requiring modification, and inform item tailoring for this caregiver population [33].

Using an electronic screening pathway that incorporates the SCNS-P&C-6 to detect the unmet needs of caregivers allows a structured, tailored approach to addressing distress and unmet needs in a clinical setting [33]. Online screening can minimize impacts on human resources, time, expenses, and burden on caregivers [28, 33]. Only those identified as having unmet needs by the general screening tool will move on to comprehensive assessment, followed by an interview with a healthcare professional for triage to clarify specific needs and recommend appropriate supportive care interventions. The ADAPT BRAINS Portal will also link to an existing online intervention for caregivers, CarersCanADAPT [37], which caregivers can choose to engage with.

As recommended [16], screening will be conducted multiple times throughout the cancer trajectory as brain tumor caregivers’ unmet needs change over time [5, 9, 12]. Given relatively few individuals were missed at each timepoint, our analysis supports the clinical utility of this screening tool. Regular rescreening will help ensure those detected as not having a need at one timepoint will be captured at subsequent screenings [33]. Additionally, as part of the ADAPT BRAINS caregiver screening pathway, caregivers will also be screened more broadly for psychological distress using the Distress Thermometer [38], General Anxiety Disorder-7 [39], and Patient Health Questionniare-9 [40]. Administering these four measures will enable holistic screening of caregivers’ psychological and supportive care needs, ensuring those with high needs requiring assistance will be detected by the pathway. Clinicians and researchers wishing to use the SCNS-P&C-6 in clinical practice settings could also consider including a general Yes/No question asking, “Do you have any other urgent needs for support that you would like to discuss with someone?” to further minimize the risk of missing caregivers with high unmet needs.

The SCNS-P&C-6 can be used beyond the Australian and ADAPT BRAINS context, enabling identification of caregivers of PwHGG requiring additional support in clinical settings globally in a timely manner. The SCNS-P&C-6 has potential to be used as a screening measure among caregivers of people with a range of cancers; however further research is needed to explore this. We recommend screening for unmet needs in caregivers of PwBT in routine care. Further research is required to establish acceptability of screening to caregivers and referral pathways for caregivers, distinguishing their needs from those of the PwBT. Integrated implementation of screening across services supporting caregivers of PwBT is essential for efficient support provision, this should include specialist cancer centres, primary care, and non-government organizations.

The original SCNS-P&C was developed to capture unmet needs in general cancer caregiver populations and contains items capturing support needs that are broad in nature. We acknowledge that caregivers of PwBT have unique needs relevant to the brain tumor experience, such as adjusting to cognitive and personality changes, and managing difficult behaviors [8]. However, the broad nature of the SCNS-P&C-6 items likely encompass these common brain tumor specific needs. Additionally, as mentioned previously, individuals identified as having some level of unmet need will proceed to more comprehensive assessment and triage to elucidate their specific needs. Our analysis was limited to caregivers of people with HGG and did not include caregivers of PwBT of other histologies and grades. Thus, further work should validate these findings in caregivers of PwBT more broadly. Planned pilot testing of the final screening items will address this and explore their suitability amongst caregivers of PwBT to ensure generalizability of the screening measure is not limited to the HGG caregiver experience.

A further limitation is that our sensitivity results are not a clinical cut-off score and should not be interpreted as such. The sensitivity results are only indicative of the extent to which a domain can potentially correctly identify individuals with unmet needs, rather than which score needs to be met for clinically significant unmet needs. Finally, as we only performed an EFA, future research should use confirmatory approaches on our brief screening measure and the original SCNS-P&C to confirm the dimensional structure of both scales.

To conclude, we psychometrically validated a brief tool to screen for the unmet needs of caregivers of people with HGG. Given the high burden associated with caring for PwBT, use of this screening tool, together with a referral pathway, has potential to reduce the negative psychological impacts of caring, and improving quality of life and the cancer experience for these caregivers.

## Electronic supplementary material

Below is the link to the electronic supplementary material.

Supplementary Material 1

Supplementary Material 2

## Data Availability

Supporting data is available on reasonable request to the corresponding author.
