# Supplementary material for: Development of a brief screening measure of unmet supportive care needs (SCNS-P&C-6) in caregivers of people with high-grade glioma
Source: J Patient Rep Outcomes. 2025 Jan 10;9:6. doi: 10.1186/s41687-024-00835-4 (PMC11723857; doi:10.1186/s41687-024-00835-4)
Supplement: Supplementary file 1 — Supplementary Material 1 [file 41687_2024_835_MOESM1_ESM.pdf]

Appendix. The SCNS-P&C-6 (final items, domains and scoring).

In the last month, what was your level of need for help with…

Item	Response options				
	No need		Some need		
	Not applicable	Satisfied	Low need	Moderate Need	High Need
Domain 1: Cancer impact needs					
1. The impact that caring for the person with cancer has had on your working life, or usual activities.	1	2	3	4	5
2. Understanding the experience of the person with cancer.	1	2	3	4	5
3. Getting emotional support for yourself.	1	2	3	4	5
Domain 2: Information and communication needs					
4. Accessing information relevant to your needs as a carer/partner.	1	2	3	4	5
5. Accessing information on what the person with cancer's physical needs are likely to be.	1	2	3	4	5
6. Having opportunities to discuss your concerns with the doctors.	1	2	3	4	5
