# Supplementary material for: Development of a brief screening measure of unmet supportive care needs (SCNS-P&C-6) in caregivers of people with high-grade glioma
Source: J Patient Rep Outcomes. 2025 Jan 10;9:6. doi: 10.1186/s41687-024-00835-4 (PMC11723857; doi:10.1186/s41687-024-00835-4)
Supplement: Supplementary file 2 — Supplementary Material 2 [file 41687_2024_835_MOESM2_ESM.pdf]

Supplementary Information: Supplementary Files

Article title: Development of a brief screening measure of unmet supportive care needs (SCNS-P&C-6) in caregivers of people with high-grade glioma.

Journal name: Quality of Life Research

Authors: Jill Chen[1], Joanne M Shaw[1], Haryana M Dhillon[1], Georgia KB Halkett[2], Emma McDougall[2], Anna K Nowak[3,4], Rachel Campbell[1]; for the BRAINs Program Investigators.

Affiliations:

1. Psycho-Oncology Cooperative Research Group, School of Psychology, Faculty of Science, The University of Sydney, Camperdown, NSW, 2006, Australia.
2. Curtin School of Nursing/ Curtin Health Innovation Research Institute, Faculty of Health Sciences, Curtin University, Bentley, WA, 6102, Australia.
3. Medical School, The University of Western Australia, Crawley, WA, 6009, Australia.
4. Department of Medical Oncology, Sir Charles Gairdner Hospital, Nedlands, WA, 6010, Australia.

Corresponding author: Dr Rachel Campbell (r.campbell@sydney.edu.au).

Supplementary File 1. Summary of final factor analysis results

Item	Rotated Component loadings		h^2
	1	2	
34. Balancing the needs of the person with cancer and your own needs.	.756	.206	.614
32. The impact that cancer has had on your relationship with the person with cancer.	.754	.111	.580
37. Getting emotional support for yourself.	.749	.262	.630
42. Making decisions about your life in the context of uncertainty.	.699	.309	.584
22. The impact that caring for the person with cancer has had on your working life, or usual activities.	.687	.221	.521
40. Dealing with others not acknowledging the impact on your life of caring for a person with cancer.	.684	.116	.481
33. Understanding the experience of the person with cancer.	.669	.234	.502
15. Looking after your own health, including eating and sleeping properly.	.649	.220	.469
38. Getting emotional support for your loved ones.	.645	.227	.468

21. Adapting to changes to the person with cancer's working life, or usual activities.	.642	.272	.486
39. Working through your feelings about death and dying.	.639	.303	.500
26. Communicating with the person you are caring for.	.579	.325	.441
44. Finding meaning in the person with cancer's illness.	.579	.113	.348
43. Exploring your spiritual beliefs.	.568	.202	.363
41. Coping with the person with cancer's recovery not turning out the way you expected.	.547	.275	.374
27. Communicating with the family.	.545	.236	.352
35. Adjusting to changes in the person with cancer's body.	.537		.369
23. Finding out about financial support and government benefits for you and/or travel insurance for the person with cancer.	.532	.393	.437
30. Handling the topic of cancer in social situations or at work.	.484	.142	.255
28. Getting more support from your family.	.454	.025	.207
29. Talking to other people who have cared for someone with cancer.	.439	.348	.314
36. Addressing problems with your sex life.	.409	-.014	.168

9. Being involved in the person with cancer’s care, together with the medical team.	.089	.846	.724
10. Having opportunities to discuss your concerns with the doctor.	.178	.801	.673
11. Feeling confidential that all the doctors are talking to each other to coordinate the person with cancer’s care.	.103	.797	.647
12. Ensuring there is an ongoing case manager to coordinate services for the person with cancer.	.145	.772	.616
7. Obtaining the best medical care for the person with cancer.	.128	.765	.601
13. Making sure complaints regarding the person with cancer’s care are properly addressed.	.188	.739	.581
8. Accessing local health care services when needed.	.244	.689	.535
6. Accessing information about the benefits and side-effects of treatments so you can participate in decision making about the person with cancer’s treatment.	.212	.683	.511
5. Accessing information on what the person with cancer’s physical needs are likely to be.	.308	.615	.473
14. Reducing stress in the person with cancer’s life.	.404	.609	.534

1. Accessing information relevant to your needs as a carer/partner.	.363	.585	.474
3. Accessing information about support services for carers/partners of people with cancer.	.408	.564	.484
2. Accessing information about the person with cancer's prognosis, or likely outcome.	.230	.553	.359
17. Addressing fears about the person with cancer's physical or mental deterioration.	.368	.550	.438
16. Obtaining adequate pain control for the person with cancer.	.100	.518	.278
**Eigenvalues**	13.87	3.52	
**% of variance explained**	37.49	9.52	

Supplementary File 2. *Domain 1: Cancer Impact Needs* two item combination R^2.

Item 1	Item 2	R^2
CNA 34 Balancing the needs of the person with cancer and your own needs	CNA 37 Getting emotional support for yourself	.639
CNA 34 Balancing the needs of the person with cancer and your own needs	CNA 42 Making decisions about your life in the context of uncertainty	.656
CNA 34 Balancing the needs of the person with cancer and your own needs	CNA 22 The impact that caring for the person with cancer has had on your working life, or usual activities	.642
CNA 34 Balancing the needs of the person with cancer and your own needs	CNA 33 Understanding the experience of the person with cancer	.556
CNA 34 Balancing the needs of the person with cancer and your own needs	CNA 15 Looking after your own health, including eating and sleeping properly	.603
CNA 34 Balancing the needs of the person with cancer and your own needs	CNA 38 Getting emotional support for your loved ones	.592
CNA 34 Balancing the needs of the person with cancer and your own needs	CNA 21 Adapting to changes to the person with cancer’s working life, or usual activities	.620

CNA 37 Getting emotional support for yourself	CNA 42 Making decisions about your life in the context of uncertainty	.624
CNA 37 Getting emotional support for yourself	CNA 22 The impact that caring for the person with cancer has had on your working life, or usual activities	.658
CNA 37 Getting emotional support for yourself	CNA 33 Understanding the experience of the person with cancer	.618
CNA 37 Getting emotional support for yourself	CNA 15 Looking after your own health, including eating and sleeping properly	.583
CNA 37 Getting emotional support for yourself	CNA 38 Getting emotional support for your loved ones	.614
CNA 37 Getting emotional support for yourself	CNA 21 Adapting to changes to the person with cancer's working life, or usual activities	.653
CNA 15 Looking after your own health, including eating and sleeping properly	CNA 38 Getting emotional support for your loved ones	.512

CNA 42 Making decisions about your life in the context of uncertainty	CNA 22 The impact that caring for the person with cancer has had on your working life, or usual activities	.632
CNA 42 Making decisions about your life in the context of uncertainty	CNA 33 Understanding the experience of the person with cancer	.506
CNA 42 Making decisions about your life in the context of uncertainty	CNA 15 Looking after your own health, including eating and sleeping properly	.573
CNA 42 Making decisions about your life in the context of uncertainty	CNA 38 Getting emotional support for your loved ones	.609
CNA 42 Making decisions about your life in the context of uncertainty	CNA 21 Adapting to changes to the person with cancer's working life, or usual activities	.628
CNA 15 Looking after your own health, including eating and sleeping properly	CNA 21 Adapting to changes to the person with cancer's working life, or usual activities	.585
CNA 38 Getting emotional support for your loved ones	CNA 21 Adapting to changes to the person with cancer's working life, or usual activities	.529

CNA 22 The impact that caring for the person with cancer has had on your working life, or usual activities	CNA 33 Understanding the experience of the person with cancer	.677
CNA 22 The impact that caring for the person with cancer has had on your working life, or usual activities	CNA 15 Looking after your own health, including eating and sleeping properly	.569
CNA 22 The impact that caring for the person with cancer has had on your working life, or usual activities	CNA 38 Getting emotional support for your loved ones	.643
CNA 22 The impact that caring for the person with cancer has had on your working life, or usual activities	CNA 21 Adapting to changes to the person with cancer’s working life, or usual activities	.518
CNA 33 Understanding the experience of the person with cancer	CNA 15 Looking after your own health, including eating and sleeping properly	.537
CNA 33 Understanding the experience of the person with cancer	CNA 38 Getting emotional support for your loved ones	.452

CNA 33 Understanding the experience of the person with cancer	CNA 21 Adapting to changes to the person with cancer’s working life, or usual activities	.564

Supplementary File 3. *Domain 2: Information and communication needs* two item combination R^2.

Item 1	Item 2	R^2
CNA 10 Having opportunities to discuss your concerns with the doctors	CNA 12 Ensuring there is an ongoing case manager to coordinate services for the person with cancer	.474
CNA 10 Having opportunities to discuss your concerns with the doctors	CNA 7 Obtaining the best medical care for the person with cancer	.547
CNA 10 Having opportunities to discuss your concerns with the doctors	CNA 6 Accessing information about the benefits and side-effects of treatments so you can participate in decision making about the person with cancer's treatment	.623
CNA 10 Having opportunities to discuss your concerns with the doctors	CNA 5 Accessing information on what the person with cancer's physical needs are likely to be	.617
CNA 10 Having opportunities to discuss your concerns with the doctors	CNA 14 Reducing stress in the person with cancer's life	.567
CNA 10 Having opportunities to discuss your concerns with the doctors	CNA 1 Accessing information relevant to your needs as a carer/partner	.581

CNA 10 Having opportunities to discuss your concerns with the doctors	CNA 3 Accessing information about support services for carers/partners of people with cancer	.646
CNA 12 Ensuring there is an ongoing case manager to coordinate services for the person with cancer	CNA 7 Obtaining the best medical care for the person with cancer	.557
CNA 12 Ensuring there is an ongoing case manager to coordinate services for the person with cancer	CNA 6 Accessing information about the benefits and side-effects of treatments so you can participate in decision making about the person with cancer's treatment	.652
CNA 12 Ensuring there is an ongoing case manager to coordinate services for the person with cancer	CNA 5 Accessing information on what the person with cancer's physical needs are likely to be	.558
CNA 12 Ensuring there is an ongoing case manager to coordinate services for the person with cancer	CNA 14 Reducing stress in the person with cancer's life	.514

CNA 12 Ensuring there is an ongoing case manager to coordinate services for the person with cancer	CNA 1 Accessing information relevant to your needs as a carer/partner	.571
CNA 12 Ensuring there is an ongoing case manager to coordinate services for the person with cancer	CNA 3 Accessing information about support services for carers/partners of people with cancer	.587
CNA 7 Obtaining the best medical care for the person with cancer	CNA 6 Accessing information about the benefits and side-effects of treatments so you can participate in decision making about the person with cancer’s treatment	.540
CNA 7 Obtaining the best medical care for the person with cancer	CNA 5 Accessing information on what the person with cancer’s physical needs are likely to be	.587
CNA 7 Obtaining the best medical care for the person with cancer	CNA 14 Reducing stress in the person with cancer’s life	.557
CNA 7 Obtaining the best medical care for the person with cancer	CNA 1 Accessing information relevant to your needs as a carer/partner	.583

CNA 7 Obtaining the best medical care for the person with cancer	CNA 3 Accessing information about support services for carers/partners of people with cancer	.628
CNA 6 Accessing information about the benefits and side-effects of treatments so you can participate in decision making about the person with cancer's treatment	CNA 14 Reducing stress in the person with cancer's life	.616
CNA 6 Accessing information about the benefits and side-effects of treatments so you can participate in decision making about the person with cancer's treatment	CNA 1 Accessing information relevant to your needs as a carer/partner	.548
CNA 6 Accessing information about the benefits and side-effects of treatments so you can participate in decision making about the person with cancer's treatment	CNA 3 Accessing information about support services for carers/partners of people with cancer	.572
CNA 5 Accessing information on what the person with cancer's physical needs are likely to be	CNA 14 Reducing stress in the person with cancer's life	.531

CNA 5 Accessing information on what the person with cancer's physical needs are likely to be	CNA 1 Accessing information relevant to your needs as a carer/partner	.598
CNA 5 Accessing information on what the person with cancer's physical needs are likely to be	CNA 3 Accessing information about support services for carers/partners of people with cancer	.485
CNA 14 Reducing stress in the person with cancer's life	CNA 1 Accessing information relevant to your needs as a carer/partner	.468
CNA 14 Reducing stress in the person with cancer's life	CNA 3 Accessing information about support services for carers/partners of people with cancer	.502

Supplementary File 4. Sensitivity vs 1-specificity results.

Coordinates of the Curve (Domain 1)

Positive if Greater Than or Equal To[a]	Sensitivity	1 - Specificity
-1.0000	1.000	1.000
4.1667	.973	.848
12.5000	.973	.727
20.8333	.926	.515
29.1667	.858	.121
37.5000	.770	.061
45.8333	.581	.000
54.1667	.446	.000
62.5000	.338	.000
70.8333	.230	.000
79.1667	.128	.000
87.5000	.047	.000
95.8333	.034	.000
101.0000	.000	.000

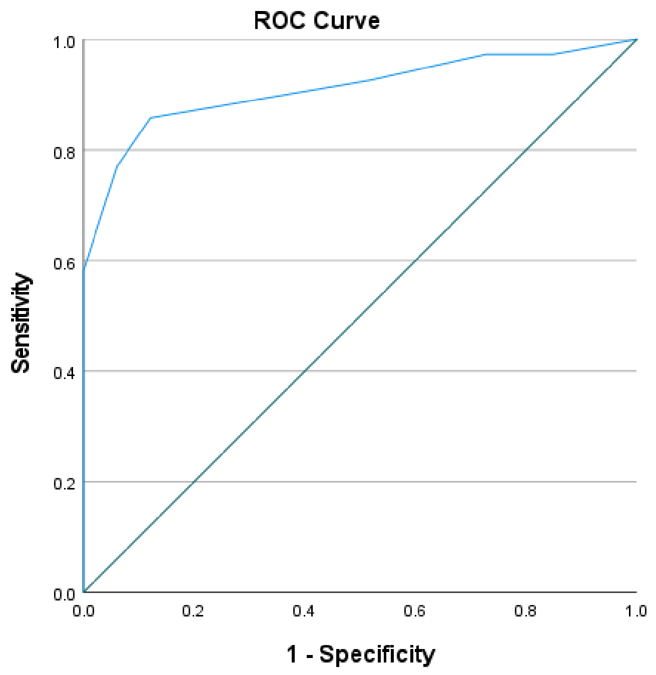

Diagonal segments are produced by ties.

Coordinates of the Curve (Domain 2)

Positive if Greater Than or Equal To[a]	Sensitivity	1 - Specificity
-1.0000	1.000	1.000
4.1667	1.000	.970
12.5000	.987	.909
20.8333	.953	.636
29.1667	.852	.091
37.5000	.725	.030
45.8333	.597	.000
54.1667	.416	.000
62.5000	.302	.000
70.8333	.168	.000
79.1667	.128	.000
87.5000	.101	.000
95.8333	.040	.000
101.0000	.000	.000

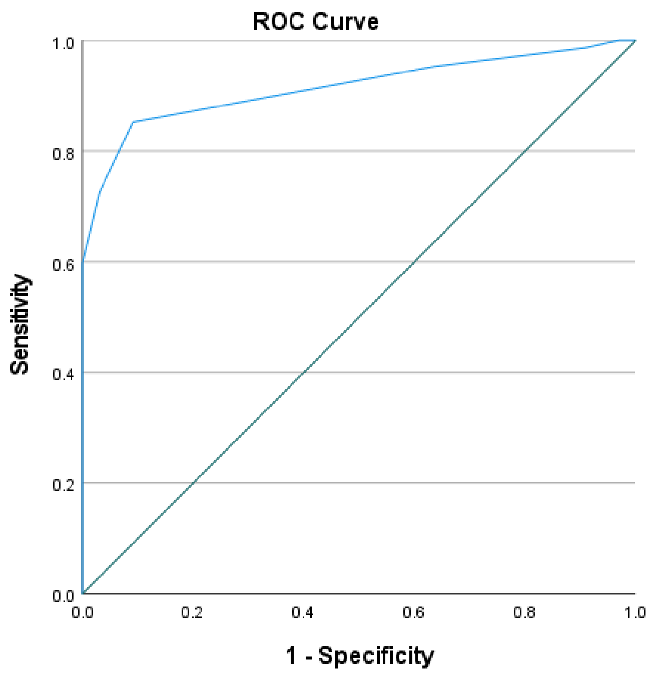

Diagonal segments are produced by ties.

Supplementary File 5. Missing needs prevalence (*N*).

		**T1 (baseline)**			**T2 (8 weeks)**		
**Original domain**	**Item**	**Low need (*N*)**	**Moderate need (*N*)**	**High need (*N*)**	**Low need (*N*)**	**Moderate need (*N*)**	**High need (*N*)**
Information needs.	CNA 2 Accessing information about the person with cancer's prognosis, or likely outcome.	3	1		1		
Information needs.	CNA 4 Accessing information about alternative therapies.		2		2		
Information needs.	CNA 6 Accessing information about the benefits and side-effects of treatments so you can participate in decision making about the person with cancer's treatment.			1			

Information needs.	CNA 23 Finding out about financial support and government benefits for you and/or travel insurance for the person with cancer.	3		1	2	1	1
Health care service needs	CNA 7 Obtaining the best medical care for the person with cancer.				1		
Health care service needs	CNA 8 Accessing local health care services when needed.				1		
Health care service needs.	CNA 11 Feeling confident that all the doctors are talking to each other to coordinate the person with cancer’s care.	1	1		2	1	
Health care service needs.	CNA 12 Ensuring there is an ongoing case manager to	1			3	2	

	coordinate services for the person with cancer.						
Health care service needs.	CNA 14 Reducing stress in the person with cancer’s life.	1	1		1		
Health care service needs.	CNA 17 Addressing fears about the person with cancer’s physical or mental deterioration.	3			1	1	
Health care service needs.	CNA 20 Finding more accessible hospital parking.				1	1	
Work and social needs.	CNA 21 Adapting to changes to the person with cancer’s working life, or usual activities.	1			2		
Work and social needs.	CNA 26 Communicating with the person you are caring for.	1			1		

Work and social needs.	CNA 27 Communicating with the family.				1		
Work and social needs.	CNA 28 Getting more support from your family.				1		
Work and social needs.	CNA 29 Talking to other people who have cared for someone with cancer.	1					
Work and social needs.	CNA 30 Handling the topic of cancer in social situations or at work.	1			2		
Psychological and emotional needs.	CNA 31 Managing concerns about the cancer coming back.	3	1		5	1	
Psychological and emotional needs.	CNA 32 The impact that cancer has had on your relationship with the person with cancer.		1		1		

Psychological and emotional needs.	CNA 34 Balancing the needs of the person with cancer and your own needs.	2			1		
Psychological and emotional needs.	CNA 35 Adjusting to changes in the person with cancer’s body.	3			1		
Psychological and emotional needs.	CNA 36 Addressing problems with your sex life.	1					
Psychological and emotional needs.	CNA 38 Getting emotional support for your loved ones.	1				1	
Psychological and emotional needs.	CNA 39 Working through your feelings about death and dying.	3			4		
Psychological and emotional needs.	CNA 41 Coping with the person with cancer’s recovery not turning out the way you expected.				1		

Psychological and emotional needs.	CNA 42 Making decisions about your life in the context of uncertainty.	2			1		
Psychological and emotional needs.	CNA 44 Finding meaning in the person with cancer’s illness.	2			1	1	
No domain.	CNA 15 Looking after your own health, including eating and sleeping properly.	1			1		
No domain.	CNA 18 Potential fertility problems in the person with cancer.	2	1	1			
No domain.	CNA 19 Caring for the person with cancer on a practical level, such as with bathing, changing dressings, or giving medications.				3		

No domain.	CNA 24 Obtaining life and/or travel insurance for the person with cancer.	2	1		2		
No domain.	CNA 25 Accessing legal services.	1			1		
